# Obsessive-Compulsive Disorder (OCD) Types and Social Media: Are Social Media Important and Impactful for OCD People?

**DOI:** 10.3390/ejihpe12080078

**Published:** 2022-08-15

**Authors:** Andrea Guazzini, Mustafa Can Gursesli, Elena Serritella, Margherita Tani, Mirko Duradoni

**Affiliations:** 1 Department of Education, Literatures, Intercultural Studies, Languages and Psychology, University of Florence, 50135 Florence, Italy; 2 Centre for the Study of Complex Dynamics, University of Florence, 50019 Sesto Fiorentino, Italy; 3 Department of Information Engineering, University of Florence, 50139 Florence, Italy

**Keywords:** obsessive-compulsive disorder, OCD, OCD types, social media, mood

## Abstract

Social media (SM) are the new standard for social interaction and people with OCD use such platforms like everyone else. However, the research on these individuals provides limited, sporadic, and difficult-to-generalize data outside of social-media evidence for one specific context concerning how SM is experienced by people with OCD. Our cross-sectional study involved 660 participants (71.4% females, 28.6% males) with 22% of the sample surpassing the 90° percentile threshold to be identified as high-level OCD-symptomatic individuals. Our work highlighted that roughly all OCD types are affected by social media in terms of mood and that these individuals appeared to give SM more importance than non-OCD individuals. The evidence presented, although very narrow, can be conceived as the first building blocks to encourage future research considering how individuals with OCD experience social media, since they appear to be affected more by them compared to non-OCD individuals.

## 1. Introduction

Obsessive-compulsive disorder (OCD) is a mental disorder where people experience intrusive thoughts and must practice rituals to relieve their discomfort [1,2]. More specifically, it would be better to refer to them as obsessive-compulsive spectrum disorders characterized by continuous mental or behavioral activity that fills most of the people’s time, with the aim of neutralizing invasive mental content [3]. All these activities are always “ego-dystonic” [3], that is, they are repugnant or inconsistent with the person’s values [4].

The characteristic symptoms of OCD are the presence of obsessions or compulsions, or both, as described by Criterion A in the *Diagnostic and Statistical Manual of Mental Disorders* [3]. Obsessions are repetitive and persistent thoughts, images, or urges that enter the mind in a recursive, intrusive, and unwanted way, causing marked distress or anxiety. Mainly for this reason, OCD patients attempt to ignore or suppress these obsessions (e.g., avoiding triggers or using thought suppression) or to neutralize them with another thought or action (e.g., performing a compulsion). Compulsions (or rituals) are repetitive behaviors or mental acts that the individual feels compelled to perform in response to an obsession; however, they are not realistically related to what they are supposed to neutralize or prevent, or are clearly excessive. Most individuals with OCD have both obsessions and compulsions [5], and they sometimes seem to have poor insight, high comorbidity, high role impairment, and a high probability of seeking treatment [6], maybe due to a specific blood flow pattern in the brain [7].

OCD diagnoses are not particularly usual but fully 28.2% of respondents to Ruscio and colleagues [6] reported experiencing obsessions or compulsions (O/C) at some time in their lives. In the *DSM-5*, between a 2–3% lifetime prevalence of OCD is reported, with equal distribution between the two sexes.

### 1.1. Types of OCD

There are multiple OCD types categorized as up-to-date subgroups of OCD that are also used as diagnostic criteria for *DSM-5* in field studies. These groups can be summarized as checking, contamination, hoarding, indecisiveness, and just right [3,8,9]. Although the types of OCD resemble each other, all of them have distinctive features that separate one from the others.

One of the most common types of subclinical OCD is hoarding, characterized by a massive collecting behavior and the consequent failure to get rid of the collected objects [6]. The *DSM-5* defines hoarding as an OCD-related disorder [3] with a persistent tendency to accumulate objects, regardless of their value, until they clutter domestic areas, making them unusable and causing psychological distress [10]. This difficulty is caused, on the one hand, by a compulsive need to store such objects, and, on the other hand, by the discomfort felt thinking of getting rid of them [3].

In the OCD type of “contamination”, obsessions and compulsions are related to both realistic and unrealistic contagions or contaminations [11,12,13,14,15]. Sometimes, the feelings of dirt are triggered even by immoral thoughts, memories of traumatic events, or mental images [16]. Of all the manifestations of OCD, the obsession with contamination is the most common in nature, and the associated cleaning compulsions are the second most common form of OCD compulsion [14].

People affected by the “checking” subtype of OCD are engaged in safety checking compulsions, with the main aim of preventing obsessive thinking related to damage, leaks, or harm to other people or oneself and to reduce uncertainty [17]. Typical examples of compulsive checking are verifying that family members are safe, repeatedly retracing the route one drove, and repeatedly checking that doors and windows are securely closed [18]. Checking is often carried out multiple times and can require hours to be completed, affecting people’s lives consistently [19].

“Indecisiveness” is defined by longer decision times as well as by increased searches for information [20], and the literature shows that it is associated with OCD tendencies and symptoms [21]. People experiencing obsessions postpone or avoid decisions in order to minimize the risk of making mistakes or not being perfect [22]. For example, indecisiveness may affect the amount of time needed for repetition of the compulsive behavior, such as the number of closings needed to actually close the door or knowing when to stop handwashing to be indeed clean [23]. OCD patients may also avoid situations when decision making is required [22] or tend to plan every aspect of their lives to reduce uncertainty [24]. For many researchers (see [25,26]), indecisiveness is only a “trait” of OCD patients, but the most used OCD Inventory [9] includes an indecisiveness dimension (i.e., sub-scale) related to OCD symptoms.

“Just right” is an OCD subtype that is characterized by uncomfortable feelings of things not being right. Patients also report feeling driven to perform an action until this uncomfortable sensation subsides, in order to feel things are “just right” [27]. The main symptoms associated with the not-just-right experiences (NJREs) are perfectionism, ordering, symmetry obsessions, compulsions, indecisiveness, and procrastination [28]. We have already addressed indecisiveness in the previous paragraph, showing that procrastination in OCD patients is due to the feeling of imperfection in their actions [22].

In the ordering and symmetry symptoms, the patient does not tolerate objects placed in a disordered or asymmetrical way, even partially. This gives them an unpleasant feeling of lack of harmony and logic [29]. Finally, NJREs are strongly linked to perfectionism [27], that is, the occurrence of NJREs increases significantly in people that experience maladaptive domains of perfectionism (e.g., concern over mistakes or doubts about actions).

### 1.2. Social Media as a Social Arena for Both Non-OCD and OCD People

In the recent decades of rapidly developing technology, social media (SM) have become an important driver for acquiring and spreading information in different domains, such as business, entertainment, science, crisis management, and politics [30,31], and they also affect psychological processes and social interactions [31].

Unfortunately, these changes are not only positive: researchers have found a new syndrome named “virtual factitious disorder” or “Munchausen by internet” [32]. These patients pretend to have online disorders to gain attention, gather sympathy, display anger, or control others with different motivations and consciousness [33]. So, technological improvements could have a critical impact on psychological diagnosis.

Furthermore, many people have started using social media as their primary source of information, thanks to the speed with which it is disseminated online [34]. Despite this, the quality of information and its truthfulness seems to be decreasing [34]. In addition to the spread of fake news, the phenomenon of fake profiles has emerged, which is the use of false credentials to create an Internet/social-media profile [35,36]. According to Facebook, 5–6% of registered accounts are fake [37].

Additionally, Good [38] identifies three shared functions of social media: (1) documenting friendship, (2) navigating new media abundance, and (3) communicating taste and building cultural capital. That is because higher levels of online engagement are due to the nature of social media such as the easy access, low costs, and fast dissemination [34]. The function of documenting friendships and collecting friends on social media could be challenging activities for a high-hoarding-scores person because these individuals are expected to collect and hoard stuff without need [39] and this feature of social media can lead them into practicing various collecting behaviors.

At this time, there are not many studies in the literature investigating the impact of SM on OCD people, and the ones we can find are fairly recent [40,41]. Even less analyzed has been the impact and the effects of social media on the specific subtypes of OCD. The only, and not compelling, evidence is reported by Luxon and colleagues [42] and concerns hoarding behaviors. The authors found that physical OCD is akin to its digital counterpart based on their study on people’s behavior on Pinterest. They displayed a positive correlation between the number of digital belongings on said platform and electronic object attachment. Some significant relationships emerged between electronic-object attachment, hoarding severity, difficulty discarding, and acquisition, and several different aspects of Pinterest use (e.g., amount of time spent on Pinterest), as well as self-reported enjoyment and importance of Pinterest. Higher levels of physical acquiring behaviors predicted increases in anxiety, fear, and anger and a decrease in relaxation and happiness after being asked to discard electronic possessions. Nevertheless, there is a gap in the literature on online hoarding behavior because it is a quite new phenomenon.

In addition, when the COVID-19 pandemic started in the last quarter of 2019, people were forced not to leave their homes for a long time [43]. The pandemic situation has increased the use of social media to more than before [44], primarily to create and maintain social bonds and contacts with friends and family [45]. The COVID-19 pandemic has also increased the time spent on online playful activities such as surfing the internet and making use of video games and SM [46,47]. Moreover, Banerjee [48] attested that, during the lockdown, hoarding behavior increased (e.g., masks, sanitizers, food, medicines). For all these reasons, the urge to study SM effects on OCD and OCD subtypes is now more important than ever. Nowadays, lots of psychologists are wondering if social media and online activities may be included in OCD symptoms. Van Bennekom and colleagues [49] propose adding obsessions, compulsions, and avoidance behavior related to new technologies (such as social media and smartphone technology) as symptoms on the Yale-Brown Obsessive-Compulsive Scale symptom checklist for OCD.

Lastly, the scientific literature stressed that individuals with higher levels of OCD symptoms may experience increased psychological distress and fatigue due to fear of missing out on social media [50]. This fear seems to push OCD people to more intense use of social media and sometimes addiction [40]. As time spent on SM increased, OCD symptoms, interpersonal sensitivity, depression, anxiety, anger–hostility, phobic anxiety, paranoid thinking, psychoticism, and additional scaly symptom levels increased too, thus affecting OCD people’s mood and well-being [51].

### 1.3. Hypotheses Development

Based on the literature knowledge provided in the previous chapter, we came up with two hypotheses to be tested with a cross-sectional and explorative research design. In the age of rapid technological developments and cheap and fast internet connections, the interaction habits of humankind have evolved. A prime example of this is social media; despite not showing much resemblance to classical social interactions, they are the most common socialization method nowadays [52]. Also, the rapid increase in social-media use [53] triggered an escalation in the number of studies focusing on social media’s impact on human psychology [54]. In this context, the majority of the studies include addiction and various psychological disorders. Unfortunately, the number of studies focusing on the interaction of sub-groups of OCD with social media is scarce and there is a research gap in this field.

The studies archived in the literature have already proven that sociodemographic variables (gender, age, etc.) can affect the adaptation to technology and social-media usage of individuals in different ways [55,56,57]. Gender is accepted into literature as a factor that affects social-media usage reasons and methods [58,59]. Another socio-demographic variable, age, is another factor in the literature that impacts individuals’ social-media usage methods and intensity [57]. Regrettably, most of the research in this area focuses on people’s adaptation to technology and the way they use social media with the concepts of age and gender. There is no research that examines the effects of these variables on the importance of social media and the social-media impacts on the mood of people in the psychological domain. Since we do not know whether age and sex really affect the way people experience social media, we decided to exploratively assess possible age and sex-related differences regarding perceived social-media importance and their impact on people’s moods, before testing our hypotheses and, thus, decide if these variables are worth being controlled as confounding variables.

In the literature, it is displayed that spending 20 min on Facebook impacts emotional state more negatively than surfing on other websites (other than social media) or not spending time on the Internet at all [60]. Meanwhile, in another study among adolescents, the positive correlation between social-media usage time and a depressed mood and anxiety is demonstrated [61]. While these exemplary studies were carried out without measuring OCD scores, the literature also showed that the moods of individuals with high OCD scores using social media were affected excessively. Furthermore, a study in the literature shows that there is a positive correlation between OCD and addictive social-media usage [40]. At the same time, it is proven in the literature that, due to fear of missing out, individuals who show some OCD symptoms control their social-media feed frequently [62,63].

**Hypothesis** **1** **(H1).** *There is a significant difference between the effects of social media on individuals’ moods whose OCD score is high compared to individuals whose OCD score is low.*

The past studies on social media, in the literature, showed us that importance of how social media affects individuals’ way of living and their psychological variables. A study performed on adolescents found that participants’ daytime sleepiness is related to their frequency of use of, and their emphasis on, social media [64]. The same study also found a significant correlation between the importance of social-media use and participants’ posting on social media and checking (apart from OCD) behavior. Also, a systematic review found mixed findings on the relationship between the importance of social media and self-injurious thoughts [65].

Advancements in technology have resulted in a change in compulsive behavior forms, making it “easier” and more “hidden” for individuals practicing these behaviors. Research on hoarding in the literature shows that people who experience hoarding problems give great importance to both the objects they collect and the compulsive behavior they perform [66,67].

Considering these, we think that compulsive social-media use may be of importance to people with a high score in OCD, just as in hoarding.

**Hypothesis** **2** **(H2).** *People with a high OCD score, on average, will report higher importance of social media than individuals with lower OCD scores*.

## 2. Methods

### 2.1. Participants

Data about the use of social media and OCD’s subtypes was collected by designing and distributing an anonymous online survey designed ad hoc through the Google Forms platform. This study was considered exempt from ethical review because it was performed upon social networks, and it did not interfere with any patient or human data beyond those strictly related to the research scopes. Moreover, account information was never collected in order to develop good research practices on social networks.

The survey was structured as 11 sections, with a total of 86 questions, on a Likert scale. The completion time was approximately 15 min. The sample was recruited through online posts on mainstream social-network sites (Facebook and Instagram, mainly). The final sample of the study was composed of 660 participants (71.4% females, 28.6% males, mean age = 24.91; standard deviation = 7.2), through a voluntary census on the web. Thus, in snowball sampling, the unbalanced gender ratio observed at the beginning was maintained until the end. To participate in the survey, participants had to self-certify to be Italian and over 18 years old.

The data were collected according to the Italian law’s requirements of privacy and informed consent (Law Decree DL-101/2018), EU regulation (2016/699), and APA guidelines. The participants had the ability to leave the session at any moment, as clearly stated in the preliminary instructions. There was no missing data, as all respondents who started the survey completed it. Data was not recorded for those participants who did not provide their informed consent or complete the survey, thus guaranteeing them the possibility of canceling any answer given up to that moment.

### 2.2. Instruments

The creation and administration of this survey engaged our team starting from June 2020 until December 2020 for a total of 6 months.

For the analysis of the symptoms and characteristics of obsessive-compulsive disorder, we referred to the Italian validation of the Vancouver Obsessional Compulsive Inventory (VOCI) [9], a scale that allowed us to evaluate the cognitive and behavioral components of OCD. It presents a total of 55 items measured on a 5-point Likert scale, divided into 6 different subscales defined in order: Checking (α = 0.96), Contamination (α = 0.92), Hoarding (α = 0.92), Indecisiveness (α = 0.85), Just Right (α = 0.89), and Obsession (α = 0.88). The set of items in the subscales showed adequate internal consistency and the total Cronbach’s α was 0.94. VOCI is validated for clinical groups with OCD, depression, or anxiety diagnosis and non-clinical individuals. Wide adoption was eased by scale translation into many languages, such as French, Turkish, Spanish, Persian, and German.

In addition, we used 2 ad-hoc items aimed to ask participants their perceived impact of social media on their mood (“Using Social Media usually affects your mood?”) and the importance they placed on social-media use (“How important do you think social media is to you?”).

### 2.3. Data Analysis and Procedure

First, prior to the participants’ recruitment, efforts were made to identify an adequate sample size for the study. Since the authors planned to use Welch’s *t*-tests (for differences between OCD and non-OCD people on continuous variables), several power analyses were performed. We relied on G*Power software to accomplish this procedure [68,69]. Power analysis allows researchers to determine the sample size required to detect an effect of a given size with a given degree of confidence. For each type of statistical analysis, a power analysis should be performed, and the final sample size should be evaluated based on the power analysis that requires the largest sample size. The adequate sample size for Chi-square was computed as a difference between two independent proportions. This power analysis showed that a sample size of 447 individuals (103 OCD people) would be enough to ensure a statistical power of 0.80, assuming a difference of around 15%, a significance level of 0.05, and a possible imbalance ratio between groups of 1:3.33. For OCD-related differences on continuous variables (*t*-test), a sample of 390 individuals would be required to reach a statistical power of 0.80, supposing a small–medium effect size (d = 0.30) and the same imbalance between groups.

To exploratively assess possible age- and sex-related differences in media variables, we planned to use Pearson correlation and *t*-test. Thus, two additional power analyses were carried out. A sample of 390 individuals would be required to reach a statistical power of 0.80, supposing a small–medium effect size (d = 0.30) and a possible sex imbalance ratio of 1:3.33. For Pearson correlation, 616 people would be enough to reach a statistical power of 0.80, supposing even a relatively small effect size (r = 0.10; [70]), and a significance level of 0.05. Since we recruited 660 people (22% with OCD total score higher than 90 percentile, 71.4% females), we considered our sample size adequate. However, given the variability of OCD-types prevalence in our sample, some of the analyses regarding OCD-related differences in continuous variables appeared slightly underpowered.

## 3. Results

### 3.1. Descriptive Statistics

As a first step, we produced descriptive statistics for the collected variables. We used mean and standard deviation for continuous variables and percentages for discrete ones. As shown in Table 1, data were presented in a sex-sensitive way (i.e., disaggregated by sex where applicable). Moreover, for each OCD type, the percentage of people over the 90° percentile threshold is indicated.

In our sample Just right, Indecisiveness, Obsession, and Contamination appeared as the most prevalent OCD types, while Checking and Hoarding are the least common.

### 3.2. Inferential Analysis

Following our hypotheses, we first checked normality (through asymmetry and kurtosis values), homoscedasticity (Breusch–Pagan test), and linearity for all the continuous variables and then assessed whether sociodemographic variables such as age and sex were related to the perceived importance and impact on mood. Males and females did not appear to differ, neither for the perceived impact of social media on their mood (t_(360.40)_ = −1.12; *p*. = 0.27), nor for the perceived importance of social media (t_(334.44)_ = −0.78; *p*. = 0.44). Instead, age showed a small but still statistically significant negative correlation with both the perceived impact of social media on mood (r = −0.17; *p*. < 0.001) and the perceived importance of social media (r = −0.18; *p*. < 0.001). Since age was only able to explain approximately 3% of social-media-related variables, we did not account for sex and age as possible confounding variables in testing subsequent hypotheses.

Following the common Cohen’s d interpretation rule [71], values of 0.2, 0.5, and 0.8 are conceived as small, medium, and large effect sizes, respectively. As shown in Table 2, for all OCD types, people with scores above the threshold reported being affected more by social media than individuals that scored below the cut-off. Notably, Hoarding and Indecisiveness types appeared to be the most affected based on Cohen’s d values, while the Just-Right type showed the smaller difference between people above and below the threshold. However, even in the case of Just Right, the magnitude of the difference appeared almost medium in size.

As we can gather from Table 3, in general, people who score high on OCD appeared to give more importance to social media than those who obtained a score below the threshold. Notably, the Hoarding type is the only type reaching a medium effect size.

Despite this, the difference between people above and below the threshold for each type of OCD on social media’s perceived importance was never negligible (i.e., it was always higher than the cut off for small effect sizes). Eventually, people with OCD appeared more like non-OCD individuals in terms of the importance conferred on social media, while they reported being more dissimilar to them in the case of the impact of SM on mood. Indeed, in the first case, the difference between people above and below the threshold was less pronounced than in the second case.

**Figure 1:**
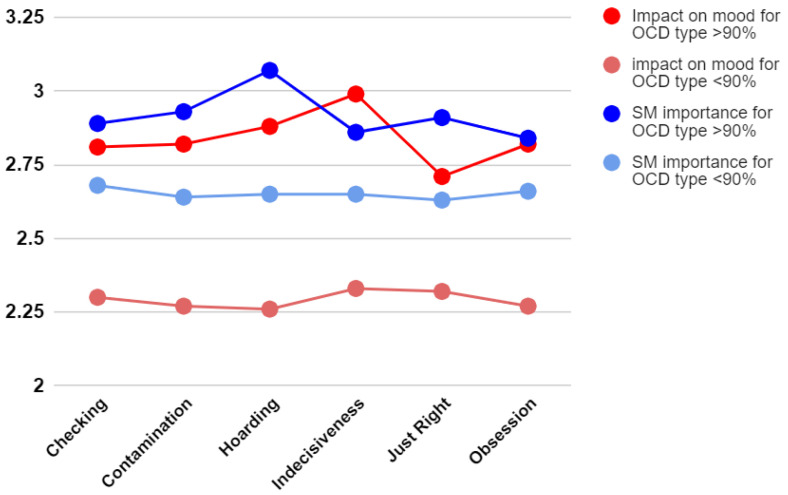
Mean values distribution across OCD types for social-media importance and social-media impact on mood.

Since both social-media importance and social-media impact on mood were single items measured on a 5-point Likert scale, we plotted Figure 1 to see how their mean values changed across OCD types.

As already highlighted by the results of the *t*-tests shown in Table 2 and Table 3, light-colored lines (referring to people who scored below the OCD threshold) are always below the red and blue lines colored more vividly, which belong to OCD people. The Hoarding type appeared to be, among the others, the OCD type that gives more importance to social media, while Indecisiveness appeared to be the most affected in terms of mood. However, none of these differences resulted as statistically significant when tested through *t*-tests, probably due to a lack of power.

## 4. Discussion

This study aimed to lay the first building blocks about how OCD people experience social media. Indeed, social media are currently an established social arena available for everyone at any time [72]. In the last decades, the literature showed how the advent of the Internet and social media has had positive consequences in various domains [30,31,34]. Unfortunately, these consequences are not only positive; there is also evidence that people could experience negative effects and consequences as a result of the use of social media, such as the emergence of new disorders related to the online world (e.g., [32]) or the worsening of previously existing conditions (e.g., [39]), such as OCD symptoms. Indeed, people with OCD showed different types of symptoms on social media. For instance, the Hoarding type of OCD triggered people to collect images from the internet and increased their importance of social media [42]. Also, previous studies showed that there is a significant relationship between checking behavior, social media, and the importance of social media [64,65].

Nonetheless, the evidence regarding OCD people and social media is sporadic, limited to a few types of OCD, and usually difficult to generalize outside one specific social media [40,41,42,50]. Further investigation on this topic should not only be welcomed but also made possible through exploratory evidence that would serve as a foundation for a new line of research. Overall, our study contributed to this by showing that roughly all OCD types are affected by social media in terms of mood (H1). Although our study did not allow us to understand the direction and the frequency of the mood change in OCD people, this result appears in line with the previous literature regarding the possible negative impact of social media on them [40,50,51]. Moreover, all types of OCD appeared to give SM more importance than non-OCD people (H2). This result may suggest, once more, that OCD people can be more exposed to undesirable outcomes, such as social-media addiction [40] or dysfunctional coping strategies [73].

Future studies should definitely mention the platforms that individuals with high OCD scores use for social media and which sub-OCD type they exhibit in their behavior. For example, previous case studies showed that individuals with high OCD scores were afraid of liking inappropriate photos on platforms such as Instagram, Facebook, and Snapchat and they continuously checked their social media [49]. On the other hand, a study carried out on Pinterest found a positive correlation between users’ digital-object-collecting behavior and hoarding behavior [42]. This proves that we cannot generalize social-media sites under the same functionalities.

Technological interventions emerge as a practical solution when social media platforms affect human psychology deeply. Users have the ability and freedom to track their time spent on social media and review the details in reports. However, in order to prevent the negative effects of social media, it is necessary to not only show reports but also inform users about the negative effects on their psychology. Educating individuals who have passed a certain amount of time on social-media platforms about the possible negative consequences can be a crucial intervention in protecting public health [74]. In addition, keeping in mind that individuals with OCD seem to be more affected by social media than non-OCD people could be a valuable aid for professionals’ choice of intervention and treatment pathways. For example, the greater importance that people with OCD symptoms place on social media could be turned into a positive resource for coping with the negative effects of the disorder, such as by implementing and using Internet-based interventions on these people. Nevertheless, several limitations of this study need to be addressed. First, the study is still explorative and cross-sectional; consequently, there is no direct evidence of causality between the variables. Participants were only Italian citizens, and we were not able to track where they inhabited which narrows the generalizability, together with the non-probability sample technique that we relied on to collect data. Eventually, criterion variables were measured through ad-hoc items (measurement bias), which is not the optimal choice in terms of validity. Eventually, we cannot exclude that some participants may have entered their answers multiple times (response bias) in good conscience, despite the efforts of the authors to invite participants to reflect upon if they had already taken part in this specific data collection.

## 5. Conclusions

Our work highlighted that high-OCD-symptoms people appeared to convey greater importance to SM and can be affected more by them in terms of mood, thus making OCD people potentially even more vulnerable to the negative side-effects of SM. In the end, we cannot ignore that OCD people use social media as everyone else and so attention should be given, and research performed on the topic, to develop (and guide them towards) inclusive and beneficial virtual environments.

## Figures and Tables

**Table 1:** Descriptive statistics of the variables included in the data collection disaggregated by sex.

		Sex		OCD Level		
Variable	Total Sample	Males	Females	OCD > 90p	OCD > 90 (Females)	OCD > 90 (Males)
	M (s.d)	M (s.d)	M (s.d)	%	%	%
OCD: Checking	2.73 (3.85)	3.07 (3.90)	2.60 (3.83)	11.2	10	14.3
OCD: Contamination	6.88 (7.26)	5.65 (6.28)	7.38 (7.58)	21.2	23.1	16.4
OCD: Hoarding	3.26 (4.27)	3.24 (4.04)	3.26 (4.36)	11.4	11.0	12.2
OCD: Indecisiveness	6.05 (4.59)	5.32 (4.11)	6.35 (4.74)	23.2	22.5	24.9
OCD: Just Right	8.64 (6.79)	8.59 (6.81)	8.66 (6.78)	24.5	24.8	23.8
OCD: Obsession	5.41 (6.52)	5.50 (6.72)	5.38 (6.44)	21.2	21.2	21.2
OCD: Total	23.98 (25.94)	31.38 (24.48)	33.62 (26.51)	22	21.4	23.3
SM impact on mood	2.41 (0.93)	2.34 (0.90)	2.43 (0.93)	-	-	-
SM importance	2.70 (0.82)	2.66 (0.85)	2.72 (0.81)	-	-	-

Note: N = 660; M = mean; s.d. = standard deviation; OCD = obsessive-compulsive disorder; SM = social media.

**Table 2:** Welch’s *t*-tests for analyzing the differences between people above and below the normative threshold regarding social media’s impact on mood for each OCD type.

Variable	Threshold	M (s.d.)	Welch’s t	df	Cohen’s d
OCD: Checking	>90°	2.81 (0.98)	−5.68 ***	202.91	0.55
	<90°	2.30 (0.88)			
OCD: Contamination	>90°	2.82 (1.00)	−6.28 ***	241.61	0.59
	<90°	2.27 (0.85)			
OCD: Hoarding	>90°	2.88 (0.96)	−7.17 ***	231.00	0.68
	<90°	2.26 (0.86)			
OCD: Indecisiveness	>90°	2.99 (1.03)	−5.25 ***	88.36	0.69
	<90°	2.33 (0.88)			
OCD: Just Right	>90°	2.71 (0.98)	−4.28 ***	205.47	0.42
	<90°	2.32 (0.89)			
OCD: Obsession	>90°	2.82 (1.06)	−3.65 ***	83.39	0.48
	<90°	2.35 (0.89)			
OCD: Total	>90°	2.88 (0.97)	−6.87 ***	212.85	0.67
	<90°	2.27 (0.86)			

Note: N = 660; M = mean; s.d. = standard deviation; OCD = obsessive-compulsive disorder; *** = *p*. < 0.001.

**Table 3:** Welch’s *t*-tests for analyzing the differences between people above and below the normative threshold regarding social media’s perceived importance for each OCD type.

Variable	Threshold	M (s.d.)	Welch’s t	df	Cohen’s d
OCD: Checking	>90°	2.89 (0.77)	−2.24 *	95.52	0.26
	<90°	2.68 (0.83)			
OCD: Contamination	>90°	2.93 (0.75)	−3.96 ***	240.11	0.37
	<90°	2.64 (0.83)			
OCD: Hoarding	>90°	3.07 (0.86)	−3.94 ***	91.42	0.50
	<90°	2.65 (0.81)			
OCD: Indecisiveness	>90°	2.86 (0.83)	−2.64 **	246.48	0.26
	<90°	2.65 (0.81)			
OCD: Just Right	>90°	2.91 (0.84)	−3.73 ***	262.07	0.34
	<90°	2.63 (0.80)			
OCD: Obsession	>90°	2.84 (0.83)	−2.29 *	218.00	0.22
	<90°	2.66 (0.82)			
OCD: Total	>90°	2.90 (0.83)	−3.34 ***	227.59	0.32
	<90°	2.64 (0.81)			

Note: N = 660; M = mean; s.d. = standard deviation; OCD = obsessive-compulsive disorder; * = *p*. < 0.05; ** = *p*. <0.01; *** = *p*. < 0.001.

## Data Availability

The data presented in this study are available on request from the corresponding author.
